# Ablation versus medication as initial therapy for paroxysmal atrial fibrillation: An updated meta‐analysis of randomized controlled trials

**DOI:** 10.1002/joa3.12641

**Published:** 2021-09-29

**Authors:** Jakrin Kewcharoen, Narut Prasitlumkum, Ronpichai Chokesuwattanaskul, Ruiyang Yi, Krit Jongnarangsin, Thomas J. Bunch, Ravi Ranjan, Leenhapong Navaravong

**Affiliations:** ^1^ Division of Cardiovascular Medicine Loma Linda University Health Loma Linda CA USA; ^2^ Division of Cardiology University of California Riverside School of Medicine Riverside CA USA; ^3^ Division of Cardiology Department of Medicine Faculty of Medicine Chulalongkorn University and King Chulalongkorn Memorial Hospital Thai Red Cross Society Bangkok Thailand; ^4^ Department of Internal Medicine John A. Burns School of Medicine Honolulu Hawaii USA; ^5^ Division of Cardiovascular Medicine Department of Internal Medicine University of Michigan Medical School Ann Arbor Michigan USA; ^6^ Division of Cardiovascular Medicine Department of Internal Medicine University of Utah School of Medicine Salt Lake City Utah USA

**Keywords:** atrial fibrillation ablation, first‐line therapy, paroxysmal atrial fibrillation

## Abstract

**Background:**

Recent randomized controlled trials (RCTs) suggest that ablation is superior to antiarrhythmic drugs (AADs) as an initial therapy for paroxysmal atrial fibrillation (pAF) to prevent arrhythmia recurrences. We performed an updated meta‐analysis of RCTs, to include recent data from cryoballoon‐based ablation and to compare arrhythmia‐free survival and adverse events between ablation and AADs.

**Methods:**

We searched MEDLINE and EMBASE from inception to December 2020. We included RCT comparing patients with pAF undergoing ablation or receiving AADs as an initial therapy. We combined data using the random‐effects model to calculate hazards ratio (HR) for arrhythmia‐free survival and odds ratio (OR) for adverse events.

**Results:**

Five studies from 2005 to 2020 involving 985 patients were included (495 patients and 490 patients underwent ablation and medication as initial therapy, respectively). Patients who underwent ablation had higher freedom from atrial tachyarrhythmias (ATs) during the 12‐24 months follow‐up period (pooled HR = 0.48, 95% CI: 0.40‐0.59, *P* < .001). In a subgroup analysis of ablation method used, both cryoablation group (pooled HR = 0.49, 95% CI: 0.38‐0.64, *P* < .001) and radiofrequency ablation group (pooled HR = 0.47, 95%CI: 0.35‐0.64, *P* < .001) showed reduction in AT recurrence compared with AAD group. There were no differences in adverse events including cerebrovascular accident, pericardial effusion or tamponade, pulmonary vein stenosis, acute coronary syndrome, deep vein thrombosis and pulmonary embolism, and bradycardia requiring a pacemaker.

**Conclusion:**

Catheter ablation (both cryoablation and radiofrequency ablation) is superior to AAD as an initial therapy for pAF in efficacy for reducing AT recurrences without a compromise in adverse events.

## Graphical abstract

We performed meta‐analysis of the most recent randomized control trials evaluating atrial fibrillation ablation as the first line therapy

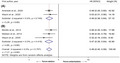

## 1 INTRODUCTION

Atrial fibrillation (AF) is the most common sustained cardiac arrhythmia affecting approximately 1%‐2% of the worldwide population.^1^ Without appropriate treatment, AF can significantly impact quality of life with risks of recurrences and/or arrhythmia progression reported to be as high as 90%.^2^, ^3^ Catheter ablation has shown to be an effective treatment with superior efficacy compared with antiarrhythmic drugs (AADs) alone for symptomatic AF.^4^, ^5^ The principal aim of AF ablation is to achieve durable circumferential pulmonary vein isolation (PVI), which electrically separates the pulmonary vein (PV) from the left atrium (LA) at the level of PV ostia/antrum. Although AF ablation is considered relatively safe, the procedure is invasive and carries risks of devastating complications such as esophageal‐related injuries, pericardial effusion with tamponade, PV stenosis, and cerebrovascular accident.^6^, ^7^ Because of this, ablation is typically used in patients who failed initial AAD therapy, and most evidence supporting the use and the superiority of ablation was derived from populations that had already received an AAD as the first‐line, rhythm‐based treatment.^8^, ^9^

Similarly, previously published randomized controlled trials (RCTs) suggest that PVI is superior to AAD even as initial therapy for paroxysmal atrial fibrillation (pAF). These include the Radiofrequency Ablation versus Antiarrhythmic Drugs as First‐Line Treatment of Paroxysmal Atrial Fibrillation (RAAFT‐2) trial, the Medical Antiarrhythmic Treatment or Radiofrequency Ablation in Paroxysmal Atrial Fibrillation (MANTRA‐PAF) trial, and the Radiofrequency Ablation versus Antiarrhythmic Drugs as First‐line Treatment of Symptomatic Atrial Fibrillation (RAAFT‐1) trial.^10^, ^11^, ^12^ A meta‐analysis of the three RCTs by Hakalathi et al confirmed this finding but reported that ablation was associated with more serious adverse events.^13^ However, all three RCTs were done using only radiofrequency ablation without any trial performing cryoablation. Moreover, the authors did not perform time‐to‐event analysis for the main outcome of arrhythmic recurrence. In this updated meta‐analysis, we included two recently published RCTs that use cryoballoon ablation, the Early Aggressive Invasive Intervention for Atrial Fibrillation (EARLY‐AF) trial and the Cryoballoon Catheter Ablation in an Antiarrhythmic Drug Naive Paroxysmal Atrial Fibrillation (STOP AF First) trial and perform a sensitivity‐analysis to compare arrhythmia‐free survival and to evaluate adverse events between the two strategies.^14^, ^15^

## 2 METHODS

### 2.1 Search strategy

Two investigators (RY and NP) independently searched for published RCTs indexed in PubMed and EMBASE from inception to December 2020 using the search terms including the following: “atrial fibrillation,” “ablation,” and “initial” as described in Supplementary file 1. Only articles in English were included. An additional manual search for potential additional pertinent studies was performed using the references from retrieved articles. Any conflict or discrepancy was resolved by a third author (LN).

### 2.2 Inclusion criteria

The inclusion criteria were as follows:

1. RCT conducted in patients with pAF comparing ablation and AAD as an initial therapy.
2. Studies must report recurrence rates of atrial tachyarrhythmias (ATs) including AF, atrial flutter and atrial tachycardia, and adverse events following the index ablation in the ablation group and in the AAD group after randomization. Hazard ratio (HR), odds ratio (OR), or sufficient raw data to calculate effect size must be provided.^16^

### 2.3 Quality of included studies

Cochrane Collaboration tool for assessing risk of bias was used to evaluate the quality of each RCT by assigning a score (high, low, or unclear) for each individual element from five domains (selection, performance, attrition, reporting, and other).^17^

### 2.4 Data extraction

A standardized data collection form was used to obtain the following data from each study including name of the first author, year of publication, country of the study, study population, main inclusion and exclusion criteria, demographic data of participants, ablation procedure details, AAD therapy, endpoint for recurrence, recurrent rates, and reported adverse event.

To ensure accuracy, this data extraction process was independently performed by all investigators. Any data discrepancy was also resolved by referring back to the original articles.

### 2.5 Statistical analysis

We performed meta‐analysis of included studies using a random‐effects model and the generic inverse‐variance method of Der Simonian and Laird to calculate pooled HR.^18^ We extracted from these studies the freedom from AT rates and complications rates. For the analysis of pooled recurrent rate, if the study does not provide HR, we manually calculate HR with methods by Tierney et al.^16^ For the analysis of adverse events, we calculate pooled OR, or if outcome was available from one study only. The heterogeneity of effect size estimates was assessed using forest plots to detect nonoverlapping confidence interval (CI) and then was calculated using the Q statistic and I^2^ statistic. For the Q statistic, substantial heterogeneity was defined as *P* **< **.10. The I^2^ statistic ranges in value from 0% to 100% (I^2^ **< **25%, low heterogeneity; I^2^ = 25%–50%, moderate heterogeneity; and I^2^ > 50%, substantial heterogeneity). A sensitivity analysis was performed to assess the influence of the individual studies on the overall results by omitting one study at a time. Publication bias was assessed using funnel plot and Egger's regression tests^19^ (*P* **< **.05 was considered significant). All statistical tests were performed using the STATA 14.2 software.

## 3 RESULTS

### 3.1 Search result

**FIGURE 1:**
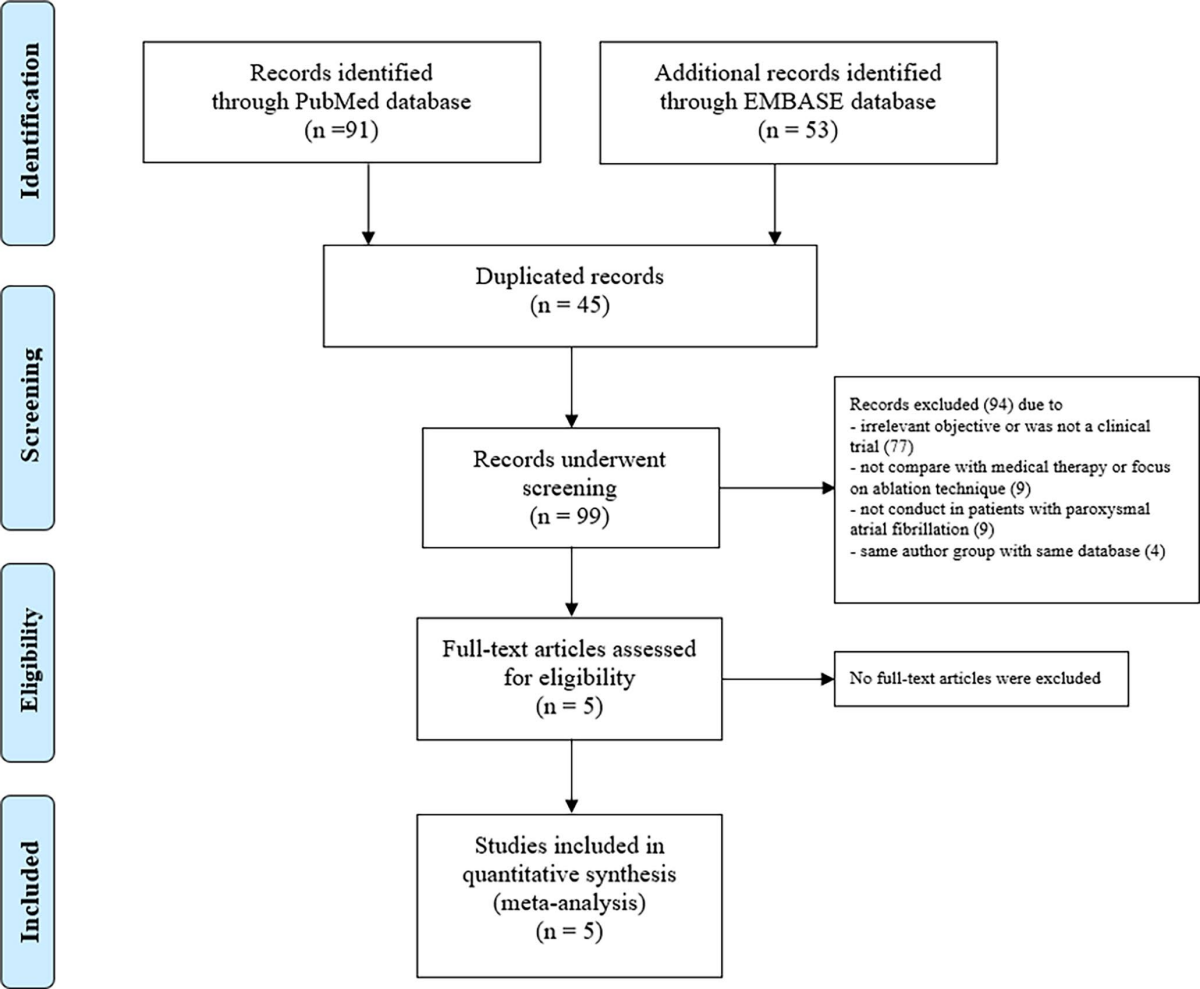
PRISMA flow diagram

Our search strategy yielded 144 potentially relevant articles (91 articles from PubMed, 53 articles from EMBASE). After the exclusion of 45 duplicate articles, 99 articles underwent title and abstract review. Furthermore, 94 articles were excluded because of at least one of the following reasons: (i) study objective was irrelevant or was not a RCT, (ii) was not conducted in patients with pAF, (iii) did not compare ablation with medical therapy or focus on an ablation technique, or (iv) same author group with the same database. This left five studies for full manuscript review. All five studies met inclusion criteria. No additional articles were added through an additional manual search. Thus, a total of five articles were included in the data analysis. The PRISMA flow diagram is shown in Figure 1.

### 3.2 Description of included studies

Five studies from 2005 to 2020 involving 985 patients were included (495 patients and 490 patients underwent ablation and medication as initial therapy, respectively).^10^, ^11^, ^12^, ^14^, ^15^ The main inclusion criterion was symptomatic pAF without previous treatment with class I or class III AADs. Exclusion criteria are reported in detail in Table 1. The most commonly used first‐line AADs were flecainide, propafenone, and sotalol, with amiodarone a second‐line AAD in all studies. AT recurrences were recorded after a blanking period of 60‐90 days and up to 1‐2 years via ambulatory cardiac monitoring. Other study characteristics of the included studies are shown in Table 2.

**TABLE 1:** Study characteristics of included randomized controlled trials

First author, trial abbreviation, year	Country	Study population/inclusion criteria	Major exclusion criteria	Analyzed participants (n)		Ablation procedure	AAD therapy	Endpoint for recurrence, follow‐up period	Recurrence at the last follow‐up (%)	
				Ablation group	AAD group				Ablation group	AAD group
Andrade, EARLY‐AF, 2020^18^	Canada	Symptomatic pAF lasting >30s without history of regular use of class I or III AADs	Previous LA ablation/surgery, reversible causes of AF, recent MI, structural or valvular heart disease, LA diameter > 5.5 cm, HF NYHA III‐IV, LVEF < 35%	154	149	Mapping: N/A Ablation: 28mm cryoballoon catheter (Arctic Front Advance, Medtronic, Minneapolis MN) Procedural endpoint: Conduction block of all PV	First‐line: flecainide (76%), propafenone (5%), sotalol (15%), dronedarone (3%)	Time to the first AT lasting >30s detected by ICM at 91 and 365 days	42.9%	67.8%
Morillo, RAAFT‐2, 2014^10^	Canada, Germany, Czech Republic, United States, Italy	Symptomatic, recurrent pAF lasting >30s and had never used AADs	Previous LA ablation/surgery, CAD, significant LVH, valvular heart disease, LA diameter > 5.5 cm, LVEF < 40%	66	61	Mapping and ablation: N/A (RF) Procedural endpoint: Conduction block of all PV	First‐line: flecainide, propafenone, sotalol, dofetilide; Second line: amiodarone	Time to the first AT lasting >30s detected by either ECG, Holter, or rhythm strip after 90‐day blanking period up to 2 years	55.4%	72.1%
Neilson, MANTRA‐PAF, 2012^11^	Denmark	Symptomatic pAF without any history of class IC and III AAD use	Previous LA ablation/surgery, HF NYHA III‐IV, LA diameter > 5 cm, LVEF < 40%, mitral valve disease, secondary causes of AF	146	148	Mapping: CARTO Ablation: RF with irrigated tip, NaviStar Thermocool (Biosense Webster, Sunnyvale, CA) Procedural endpoint: Elimination of all electrical activity >0.2 mV	First‐line: flecainide, propafenone; Second line: amiodarone	Time to first any AF lasting >60s detected by Holter after 90‐day blanking period up to 2 years	15.0%	28.8%
Wazni, RAAFT‐1, 2005^12^	United States, Italy, Germany	Symptomatic pAF without any history of AAD use	Previous LA ablation or any open‐heart surgery	33	37	Mapping: N/A Ablation: RF, 8‐mm tip ablation catheter (Biosense Webster, Baldwin Park, Calif, and EP Technologies, Sunnyvale, Calif) Procedural endpoint: electrical disconnection of the PV antrum from LA	First‐line: flecainide, propafenone, sotalol; Second line: amiodarone	Time to first any AF lasting >15s detected by Holter between 60 days to 1 year	15.2%	68.3%
Wazni, STOP AF First, 2020^19^	United States	Symptomatic pAF without history of class I and III AAD use for >7 days	History of any cardiac surgery, HF NYHA III‐IV, LA diameter>5 cm, LVEF < 45%, valvular heart disease, secondary causes of AF	104	99	Mapping: N/A Ablation: 2nd generation cryoballoon catheter (Arctic Front Advance, Medtronic, Minneapolis, MN) Procedural endpoint: entrance block of all PV	flecainide (61%), propafenone (7%), sotalol (7%), dronedarone (12%), amiodarone (2%)	Time to the first AT lasting >30s detected by ambulatory monitoring or >10s detected by 12‐lead ECG after 90‐day blanking period up to 1 year	20.2%	35.4%

Abbreviations: AADs, antiarrhythmic drugs; AF, atrial fibrillation; AT, atrial tachyarrhythmia; HF, heart failure; LA, left atrium; LVEF, left ventricular ejection fraction; NYHA, New York Heart Association; pAF, paroxysmal atrial fibrillation; PVI, pulmonary vein isolation.

**TABLE 2:** Basic characteristics of study participants

First author, trial abbreviation, year	Age (years)		Male (%)		CHA2DS2‐VASc score^a^		HTN (%)		DM (%)		CAD (%)		Anticoagulation (%)		Baseline episodes of pAF		LVEF (%)		LA size (mm)	
	Ablation group	AAD group	Ablation group	AAD group	Ablation group	AAD group	Ablation group	AAD group	Ablation group	AAD group	Ablation group	AAD group	Ablation group	AAD group	Ablation group	AAD group	Ablation group	AAD group	Ablation group	AAD group
Andrade, EARLY‐AF, 2020^18^	57.0.7 ± 12.3	59.5 ± 10.6	72.7%	68.5%	Mean 1.9 ± 1.0	Mean 1.9 ± 1.1	37%	36.9%	N/A	N/A	7.8%	4.7%	Warfarin 3.2%, Non–vitamin K antagonist 63.6%	Warfarin 6.0%, Non–vitamin K antagonist 58.4%	Median 3/month (1‐10)	Median 3/month (1‐10)	Mean 59.6 ± 7.0%	Mean 59.8 ± 7.6%	Mean 39.5 ± 5.0 mm	Mean 38.1 ± 6.5 mm
Morillo, RAAFT‐2, 2014^10^	56.3 ± 9.3	54.3 ± 11.7	77.3%	73.8%	Mean 0.5 ± 0.7	Mean 0.7 ± 0.8	42.4%	41.0%	1.5%	6.6%	9.1%	3.3%	53.0%	31.1%	Median 10 in the past 6 months (5‐40)	Median 10 in the past 6 months (4‐40)	Mean 61.4 ± 4.8%	Mean 60.8 ± 7.0%	Mean 40 ± 5 mm	Mean 43 ± 5 mm
Neilson, MANTRA‐PAF, 2012^11^	56 ± 9	54 ± 10	68%	72%	≥2:18.4%	≥2:19.6%	29%	36%	4%	7%	4%	1%	N/A	N/A	N/A	N/A	40%‐60%: 19.8%, >60%: 79.4%	40%‐60%: 17.5%, >60%: 81.7%	Mean 40 ± 6 mm	Mean 40 ± 5 mm
Wazni, RAAFT‐1, 2005^12^	53 ± 8	54 ± 8	N/A	N/A	N/A	N/A	N/A	N/A	N/A	N/A	N/A	N/A	N/A	N/A	Mean duration 5 ± 2 months	Mean duration 5 ± 2.5 months	Mean 53 ± 5%	Mean 54 ± 6%	Mean 41 ± 8 mm	Mean 42 ± 7 mm
Wazni, STOP AF First, 2020^19^	60.4 ± 11.2	61.6 ± 11.2	61%	58%	≥2:55%	≥2:55%	56%	58%	14%	17%	12%	12%	69%	69%	N/A	N/A	Mean 60.9 ± 6.0%	Mean 61.1 ± 5.9%	Mean 38.7 ± 5.7 mm	Mean 38.2 ± 5.4 mm

Abbreviations: AADs, antiarrhythmic drugs; AF, atrial fibrillation; CAD, coronary artery disease; DM, diabetes mellitus; HTN, hypertension; LA, left atrium; LVEF, left ventricular ejection fraction; N/A, not applicable; pAF, paroxysmal atrial fibrillation.

^a^ CHA2DS2‐VASc scores: a clinical estimation of the risk of stroke among patients with atrial fibrillation with higher scores indicating a greater risk of stroke

### 3.3 Quality assessment of included studies

The Cochrane Collaboration tool for assessing risk of bias is shown in Table 3.

**TABLE 3:** Cochrane collaboration tool to assess risk of bias for randomized controlled trials

First author, year	Selection bias		Performance bias	Detection bias	Attribution bias	Reporting bias	Other bias	Overall bias risk
	Random sequence generation	Allocation concealment						
Andrade, 2020^18^	Low	Low	Low	Low	Low	Low	Low	Low
Morillo, 2014^10^	Low	Low	Low	Low	Low	Low	Low	Low
Neilson, 2012^11^	Low	Low	Low	Low	Low	Low	Low	Low
Wazni, 2005^12^	Low	Low	Low	Low	Low	Low	Low	Low
Wazni, 2020^19^	Low	Low	Low	Low	Low	Low	Low	Low

### 3.4 Meta‐analysis results

#### 3.4.1 Freedom from atrial tachyarrhythmia

**FIGURE 2:**
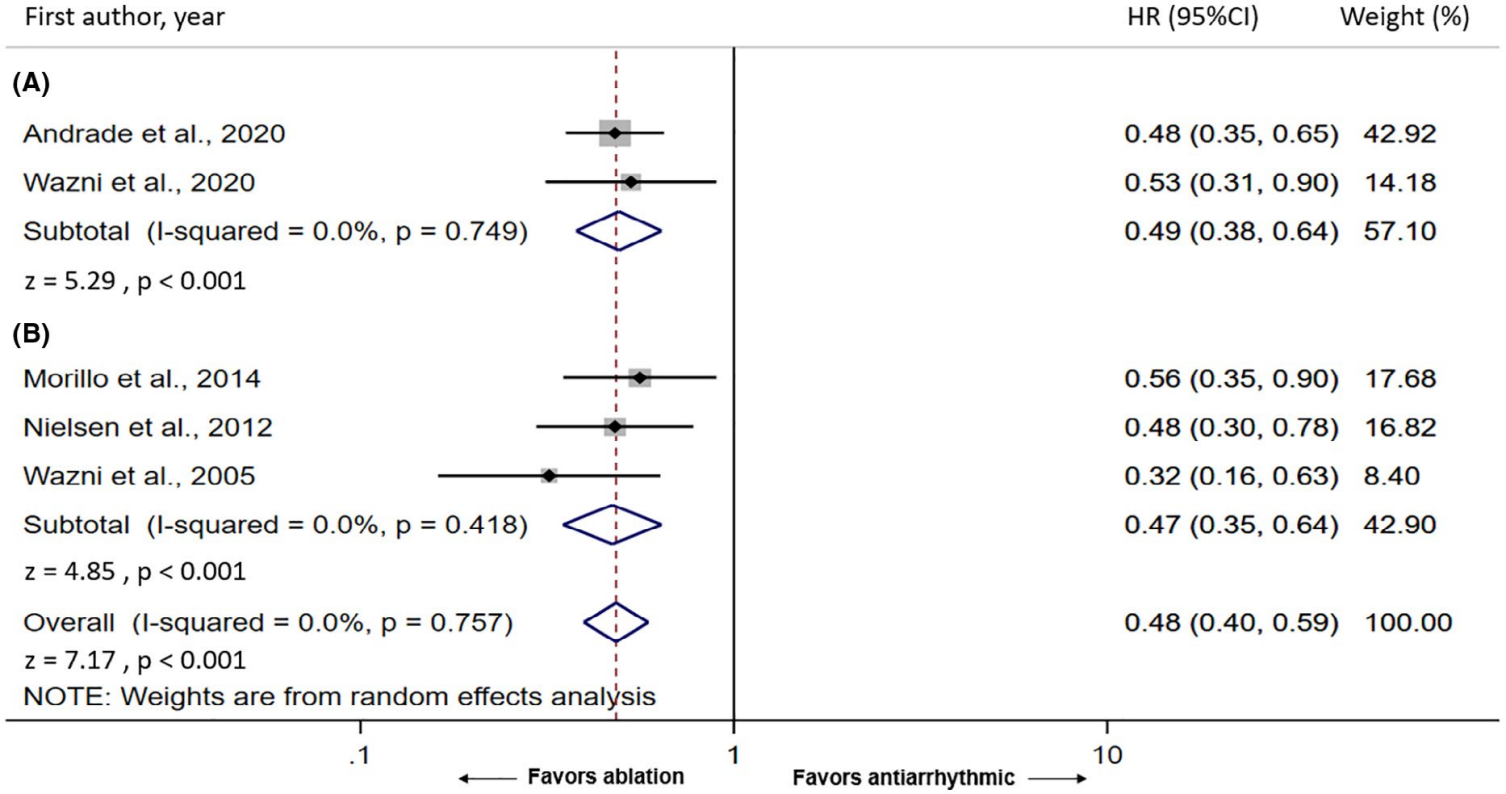
Forest plot of the included studies comparing arrhythmia‐free survival between ablation group and antiarrhythmic medication group; 2A: cryoablation, 2B: radiofrequency ablation

The outcome of AT recurrences was available in all five studies.^10^, ^11^, ^12^, ^14^, ^15^ Patients who underwent catheter ablation for PVI had an increased freedom from AT during the 12‐24 months of follow‐up period (pooled HR = 0.48, 95% CI: 0.40‐0.59, *P* < .001, I^2^ = 0.0%) compared with AAD therapy (Figure 2). In the subgroup analysis of ablation method, patients in the cryoablation group (pooled HR = 0.49, 95% CI: 0.38‐0.64, *P* < .001, I^2^ = 0.0%) (Figure 2A) and radiofrequency ablation group (pooled HR = 0.47, 95% CI: 0.35‐0.64, *P* < .001, I^2^ = 0.0%) (Figure 2B) both had increased freedom from AT during the 12‐24 months of follow‐up period compared with AAD therapy.

#### 3.4.2 Adverse events

**FIGURE 3:**
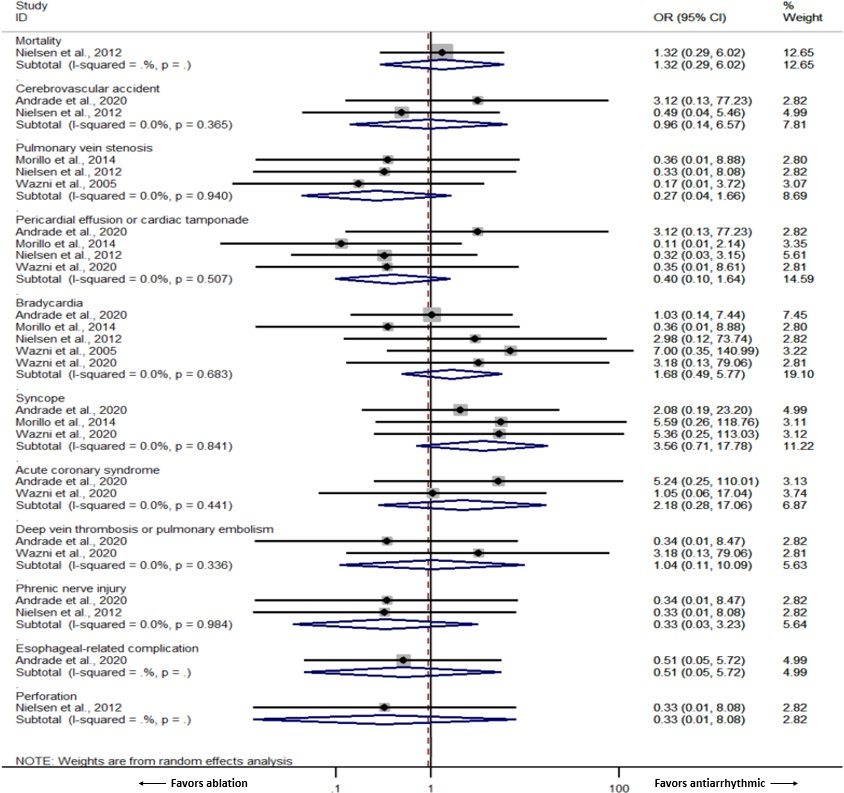
Forest plot of the included studies demonstrating available adverse events

A summary of adverse events from the included studies is shown in Table 4. Studies reporting zero event rates in both ablation and AAD group were excluded from the meta‐analysis for adverse events. As such, we performed a meta‐analysis of adverse events with these available outcomes in at least two of the included studies. There were no differences in adverse events from studies in which data were available for meta‐analysis for cerebrovascular accident, PV stenosis, pericardial effusion or tamponade, bradycardia requiring pacemaker, syncope, acute coronary syndrome, deep vein thrombosis (DVT), and pulmonary embolism (PE). Forest plot and table summarizing adverse events are shown in Figure 3 and Table 4, respectively.

**TABLE 4:** Summary of adverse events

Adverse events	Studies reporting the adverse events	Event in ablation group	Event in CBA	Event in RFA	Event in AAD group	Studies available for analysis	OR (95%CI) or pooled OR (95%CI)^a^
Mortality	3	3/366	0/154	3/212	4/358	1	0.76 (0.17 to 3.44)
Cerebrovascular accident	4	2/398	0/154	2/244	2/393	2	1.05 (0.15‐7.19)
Pulmonary vein stenosis	3	4/244	N/A	4/244	0/244	3	3.74 (0.60‐23.26)
Pericardial effusion or cardiac tamponade	4	8/470	1/154	7/316	2/457	4	2.50 (0.61‐10.26)
Bradycardia	5	3/502	2/258	1/244	7/492	5	0.59 (0.17‐2.04)
Syncope	3	1/324	1/258	0/66	6/309	3	0.28 (0.06‐1.40)
Acute coronary syndrome	2	1/258	1/258	N/A	3/248	2	0.46 (0.06‐3.57)
Deep vein thrombosis or pulmonary embolism	3	1/290	1/258	0/32	1/283	2	0.96 (0.10‐9.29)
Hematoma	2	2/300	1/154	1/146	0/297	2	2.99 (0.31‐28.92)
Phrenic nerve injury	1	3/154	3/154	N/A	0/149	1	6.91 (0.35‐134.88)
Esophageal‐related complication	2	2/220	2/154	0/66	1/210	1	1.95 (0.17‐21.71)
Perforation	1	1/146	N/A	1/146	0/148	1	3.06 (0.12‐75.78)

Abbreviations: AADs, antiarrhythmic drugs; CBA, cryoballoon ablation; CI, confidence interval; RFA, radiofrequency ablation; N/A, not applicable; OR, odds ratio.

^a^ Odds of developing the complication in the ablation group (both CBA and RFA) compared with the AAD group.

### 3.5 Publication bias

We aimed to investigate potential publication bias via the funnel plot and Egger's test. However, as we only had up to five studies in the main analysis (Figures 1 and 2), the number was insufficient to reject the assumption of no funnel plot asymmetry. Thus, we did not perform a funnel plot or Egger's test.^20^, ^21^

## 4 DISCUSSION

The main finding from our updated study that includes contemporary ablation tools is that catheter ablation for PVI is more effective than AAD as initial therapy for pAF in reducing AT recurrences after the initial blanking period to 1‐2 years. In addition, this benefit with ablation was the same in both cryoablation and radiofrequency ablation with similar relative reductions in risks. Despite the augmented efficacy for reduction in AF recurrences with catheter ablation, we did not observe a significant increase in any adverse events in these patients compared with AAD therapy.

In this updated meta‐analysis of RCTs, we found two recently published RCTs that add to prior summary work in this area. The new search resulted in a total of five RCTs to date. The two newly added RCTs, EARLY‐AF, and STOP AF First, used cryoballoon ablation for PVI, which is different from the previous three RCTs that all used radiofrequency ablation. Evidence from the Cryoballoon or Radiofrequency Ablation for Paroxysmal Atrial Fibrillation (FIRE AND ICE) trial suggested that treatment efficacy of cryoballoon ablation was noninferior to the more traditional radiofrequency ablation in drug‐refractory pAF.^22^ However, the efficacy of cryoablation in treatment‐naïve pAF was still unclear, especially when compared directly with AAD or radiofrequency ablation. The EARLY‐AF trial and the STOP AF First trial were the first RCTs to compare cryoablation with AAD as an initial therapy for pAF. This gave us opportunity to indirectly compare the two ablation strategies as initial treatment for pAF. As shown in Figure 1, the pooled HR for the cryoablation subgroup (Figure 1A) was similar to the pooled HR for the radiofrequency ablation subgroup (Figure 2). However, as these pooled data are derived from a retrospective comparison, further prospective RCTs that directly comparing the two techniques are needed to confirm this finding or investigate if one may be preferable over the other for treatment naïve pAF compared with AAD.

The included studies in our meta‐analysis demonstrated a significant reduction in AT recurrence at 1‐2 years with a similar effect size. The EARLY‐AF trial and the RAAFT‐2 trial reported HRs for AT recurrence. In the MANTRA‐PAF trial, the RAAFT‐1 trial, and the STOP‐AF First trial, the authors did not report HRs for AT recurrence. Nevertheless, the authors provided sufficient raw data for us to calculate HRs as described in the methods section. The HRs from each study are shown in Figure 1 along with the pooled HR.

The study designs are similar among the five included RCTs. Inclusion and major exclusion criteria are shown in Table 1. Briefly, all studies included strictly symptomatic pAF without prior use of class I and III AADs. Major exclusion criteria being used in all studies were previous ablation or surgery of the LA, reversible causes of AF, structural or valvular heart disease, a left atrial diameter >5‐5.5 cm, a left ventricular ejection fraction <35%–45%, NYHA class III‐IV heart failure. For AT outcome measurements, all studies reported using a blanking period of 60‐90 days following the ablation with follow‐up of patients up to 1‐2 years. Three studies used radiofrequency ablation, and two studies used cryoablation as described in Table 1. The subgroup analysis by ablation method is shown in Figure 2A,B that demonstrates similar results between the two ablation techniques. The consistency and similarity in study design likely contributed to the absence of heterogeneity (I = 0.0%) in our analysis, which indicates that our result is robust.

Atrial arrhythmia type as endpoint differs slightly among the included studies. The MANTRA‐PAF trial and the RAAFT‐1 trial considered only AF as the clinical endpoint for recurrence. For the EARLY‐AF trial, the RAAFT‐2 trial, and the STOP AF First trial, authors consider either of AF, atrial flutter, and atrial tachycardia as the clinical endpoint. It is likely because of the difference in definition of endpoint that the MANTRA‐PAF trial and the RAAFT‐1 trial reported lower recurrence rates than the other 3 RCTs, except for the AAD group from the RAAFT‐1 trial that had a comparable recurrent rate with the other three RCTs.

Table 4 shows a summary of the adverse events. Overall, the adverse event rates were low, and not many specific adverse events of interest occurring in either of the ablation group or AAD group in some studies. We were able to perform a meta‐analysis on several adverse events including cerebrovascular accident, PV stenosis, pericardial effusion or tamponade, bradycardia requiring pacemaker, syncope, acute coronary syndrome, DVT or PE, all of which were statistically similar between the two groups. For other adverse events including mortality, phrenic nerve injury, esophageal‐related complications, and perforation, there was only one study available for a meta‐analysis for each outcome. The individual ORs for these adverse events also did not demonstrate significant differences between the two groups. Nevertheless, these findings must be interpreted with caution as this could be from inadequate power from the extremely low number of events which subjects this sub‐analysis to risk of a type II error.

## 5 LIMITATIONS

We acknowledge certain limitations within our study. First, extracted/calculated HRs were not adjusted for confounders. Second, there were differences in follow‐up times with three and two studies that followed patients up to 12 and 24 months, respectively. Also, there was a difference in the definition of arrhythmia recurrences used for the clinical endpoint as discussed above. Nevertheless, we did not observe major differences in the HR, and the random‐effect model did not reveal significant heterogeneity from our analysis (I^2^ = 0.0%). Third, data regarding the adverse event were limited because of the overall low event rates which subsequently limited the power of the analysis.

## 6 CONCLUSIONS

In this updated systematic review and meta‐analysis of RCTs, that now includes RCTs that specifically use cryoballoon ablation only, we found that catheter ablation is more effective than AADs as an initial therapy for pAF in reducing AT recurrences over 1‐2 years following the treatment initiation. These results are the same with use of both cryoablation and radiofrequency ablation approaches. The adverse event rates are low with contemporary use of AADs and with evolved catheter ablation tools, and there were no differences in any adverse events between the two groups.

## CONFLICT OF INTEREST

All authors declare no conflict of interest.

## Supporting information

Supplementary Material
