# Supplementary material for: Ablation versus medication as initial therapy for paroxysmal atrial fibrillation: An updated meta‐analysis of randomized controlled trials
Source: J Arrhythm. 2021 Sep 29;37(6):1448–58. doi: 10.1002/joa3.12641 (PMC8637078; doi:10.1002/joa3.12641)
Supplement: Supplementary file 1 — Supplementary Material [file JOA3-37-1448-s001.docx]

MedLine

(("atrial fibrillation"[MeSH Terms] OR ("atrial"[All Fields] AND "fibrillation"[All Fields]) OR "atrial fibrillation"[All Fields]) AND ("ablate"[All Fields] OR "ablated"[All Fields] OR "ablates"[All Fields] OR "ablating"[All Fields] OR "ablation"[All Fields] OR "ablational"[All Fields] OR "ablations"[All Fields]) AND ("initial"[All Fields] OR "initially"[All Fields] OR "initials"[All Fields] OR "initiate"[All Fields] OR "initiated"[All Fields] OR "initiates"[All Fields] OR "initiating"[All Fields] OR "initiation"[All Fields] OR "initiations"[All Fields] OR "initiator"[All Fields] OR "initiators"[All Fields])) AND (randomizedcontrolledtrial[Filter])

EMBASE

('atrial fibrillation ablation initial' OR (atrial AND ('fibrillation'/exp OR fibrillation) AND ablation AND initial)) AND ([article]/lim OR [article in press]/lim) AND [randomized controlled trial]/lim
